# LATINO COMMUNITY ENGAGEMENT PROJECTS FOR OLDER ADULTS LED BY LATINO NURSING STUDENTS AND FACULTY

**DOI:** 10.1093/geroni/igae098.1582

**Published:** 2024-12-31

**Authors:** Jung-Ah Lee

**Affiliations:** University of California Irvine, Irvine, California, United States

## Abstract

The Latino population in California has experienced a continual increase in recent decades. As of 2022, the United States Census Bureau estimated that 15.73 million Latinos resided in California, constituting 39.4% of the state’s total population. This presentation aims to introduce ongoing community engagement projects conducted by bilingual Spanish/English speaking nursing students and faculty, and their impact on the health of Latino older adults and their families in Southern California. The school of nursing is situated in major cities housing the state’s largest Latino communities, with compassionate community engagement as one of its core missions. The nursing faculty has consistently developed community-based projects through the curriculum and research, providing extensive one-on-one mentoring to groups of Latino students for several years. Key initiatives targeting the Latino older adult population include: (1) Nursing Skill Education Sessions for In-Home Supportive Service (IHSS) Caregivers, offering essential care to Medicaid older adults with chronic conditions, (2) In-home caregiving education for Latino family caregivers of people living with dementia (PWD), and (3) Telephone support projects for isolated Latino caregivers of PWD during the pandemic crisis. Under the close supervision of faculty with geriatric expertise, Spanish-speaking nursing students actively engaged with Latino older adults and their families through the projects. Spanish-speaking IHSS caregivers appreciated the hands-on learning opportunities in nursing skills with Spanish educational materials. Participating students expressed their compassion to serve and support the community. Continued emphasis on Latino community-centered education and research projects is essential to meet the evolving needs of the community.

